# Isolation and identification of a phosphate solubilising fungus from soil of a phosphate mine in Chaluse, Iran

**DOI:** 10.1080/21501203.2016.1221863

**Published:** 2016-08-23

**Authors:** Raheleh Jamshidi, Bahi Jalili, Mohamad Ali Bahmanyar, Soroosh Salek-Gilani

**Affiliations:** Department of Soil Science, Sari University of Agricultural Sciences and Natural Resources, Sari, Iran

**Keywords:** Phosphate-solubilising fungi, Molecular identification, Rock phosphate, P solubilisation

## Abstract

Microbial solubilisation of phosphorus from insoluble phosphates is an environmental friendly and cost effective approach in sustainable soil management. Introducing the indigenous microorganisms to soil requires shorter adaptation period and causes fewer ecological distortions than exogenous microorganisms. This study was conducted to isolate and identify the indigenous fungi for phosphate solubilisation in Mazandaran, Iran. A potent phosphate solubilising fungus was isolated from an Iranian phosphate mine and selected for solubilisation of rock phosphate (RP). The identified fungus was characterised by calmodulin-based polymerase chain reaction method as *Aspergillus tubingensis* SANRU (Sari Agricultural Sciences and Natural Resources University). The phosphate solubilisation ability of the fungal strain was carried out in shake-flask leaching experiments containing various concentrations of RP (1%, 2%, 4%, or 8% w/v). The maximum P solubilisation rate of 347 mg/l was achieved at 1% of RP concentration on day 9. The regression analysis indicated that the P solubilised mainly through acidification. This study shows the possibility of using *A. tubingensis* SANRU for application in the management of P fertilisation.

## Introduction

Phosphorus (P) is an essential plant nutrient that plays a vital role in energy transfer reactions, respiration, macromolecular biosynthesis, and photosynthesis (Kuhad et al. 2011). Most soils around the world are deficient in P because a large portion of soil P is being ﬁxed as insoluble phosphates of Al, Ca, or Fe, therefore it is not available to plant as a nutrient source (Gyaneshwar et al. 2002). Hence, application of commercial P fertilisers is required to obtain good productivity in most soils. Acidification of rock phosphates (RPs) with strong acid is a common method for production of phosphate fertilisers; however, it is a costly process. The generous use of P fertilisers, on the other hand, leaches to the ground water and leads to water eutrophication (Singh and Reddy 2011). In recent years, microbial solubilisation of RPs has gained increasing attention, since this approach is simple, economic, and environmental friendly. (Chai et al. 2011). RP consists mainly of apatite. The general formula of apatite is Ca_5_(PO_4_)_3_(OH,F,Cl) depending on the last functional group. It is referred to as hydroxy-, ﬂuoro-, or chloro-apatite (Hughes and Rakovan 2002). The most common gangue minerals in RPss are quartz, chert, clay, feldspar, mica, calcite, and dolomite (Heydarpoura et al. 2011). Bio-solubilisation of RPs is based on the ability of autotrophic and heterotrophic microorganisms to recover P from insoluble phosphate compounds. The autotrophic solubilisation of RP is based on sulfuric acid production by *Acidithiobacillus ferrooxidans* and *Acidithiobacillusthiooxidans* in the presence of sulfur source (Bhatti and Yawar 2010). The heterotrophic solubilisation of RP is done using heterotrophic bacteria (especially genera *Bacillus* and *Pseudomonas*) and fungi (genera *Penicillium* and *Aspergillus* (*A*)). Among the P-solubilising microorganisms, filamentous fungi, especially black *Aspergilli* including *A. aculateus, A. awamori, A. niger, A. tubingensis*, as well as *Penicillium* have shown potential for solubilisation of insoluble P compounds (Achal et al. 2007). The phosphate solubilising ability of fungi has been accompanied with acidification, complexolysis, and acid phosphatase production.

The present work focuses on isolation and identification of potent filamentous fungi to solubilise RP under *in vitro* conditions from Dalir phosphate mine site, which is located in Chaluse, Iran.

## Materials and methods

### Rock phosphate

The RP samples were collected from the Esfordi alkali-magmatic phosphate mine, Bafgh, Iran. The samples were pulverised and sieved in order to 60 µm particle size fraction passes through for mineralogical, elemental, and bioleaching experiments. Mineralogical and elemental composition of RP was determined by using X-ray diffraction (XRD; GNR MPD3000) analysis and X-ray ﬂuorescence (XRF; ARL 8410), respectively. For XRD analysis, a small amount of RP sample (0.1 g) was placed into a percussion mortar, 10 ml deionised water was added, and the sample was grounded using pestle. The slurry was transferred to glass slides and then the air dried slide was placed on the XRD meter for scanning using GNR-MPD 300 (GNR Analytical Instruments Group, Novara, Italy) with Cu*K*_α_ radiation with the wavelength of 15,418 Å. Elemental composition of RP sample was determined by using XRF under a vacuum atmosphere.

### Screening for phosphate solubilising fungi

Mine soil samples were collected from Dalir phosphate and Esfordi alkali-magmatic mine sites. Over 25 random points were chosen and samples (2 kg) were collected from the upper 10 cm layer of each point. All materials were thoroughly mixed and one-third of each sample was stored at 4°C prior to carrying out the microbial experiments. Two-thirds of each sample was air dried for 3 days, sieved through 2 mm mesh, homogenised, and stored in plastic bags prior to their use. Preceding the microbial experiments, soil samples were thoroughly mixed and 10 g of each was placed in a 250-ml Erlenmeyer flask containing 95 ml of 0.1 M (NH_4_)_2_HPO_4_ buffer and shaken at 25°C for 30 min, serially diluted in triplicate. An aliquot (100 µl) of the solution was aseptically spread on National Botanical Research Institute’s Phosphate (NBRIP) growth agar medium (Nautiyal 1999) containing 10 g/l glucose, 5 g/l Ca_3_(PO_4_)_2_, 5 g/l MgCl_2_·6H_2_O, 0.25 g/l MgSO_4_·7H_2_O, 0.2 g/l KCl, 0.1 g/l (NH_4_)_2_SO_4_, and 1.5% agar supplemented by bromophenol blue. Seven days post incubation at 28 ± 2°C, colonies with distinct clear halo zones were further purified by replanting on NBRIP medium. Among the fungal isolates, the ones that produced the biggest halo zone (mm) after 48 h were selected for further studies.

### Molecular identification of fungal isolate

#### Culture of fungal mycelia

A volume of 100 ml potato dextrose broth was inoculated with one colony of the fungus grown on potato dextrose agar (PDA) for 2–3 weeks and incubated at 28°C for an additional week with shaking at 250 rpm. The mycelium was harvested by centrifugation at 4000 rpm for 30 min and then filtered through Whatman filter paper, washed with autoclaved distilled water, and freeze-dried in liquid nitrogen.

#### Extraction of fungal DNA

Freeze-dried mycelium was ground to a fine powder using a mortar and pestle in liquid nitrogen. Immediately, DNA was extracted using the DNeasy Plant Mini Kit according to the manufacturer’s instructions (69104; Qiagen). In brief, 100 mg of the ground mycelium was suspended and lysed in 450 µl of lysis buffer (AP1) and RNase A. The mixture was incubated for 10 min at 65°C. An amount of 130 µl of Buffer AP2 was added to the lysates, mixed, and incubated for 5 min on ice. Lysate was centrifuged for 5 min at 20,000 × *g* and then applied to the QIAshredder Mini Spin Column and centrifuged for 2 min at the same speed. Flow-through fraction was mixed with 1.5 volumes of Buffer AP3, mixed, applied to the DNeasy Mini Spin Column, and centrifuged for 1 min at 6000 × *g*. DNeasy Mini Spin Column was washed with 500 µl of Buffer AW and centrifuged for 2 min at 20,000 × *g*. Genomic DNA (gDNA) was eluted from the DNeasy membrane by adding 100 µl of Buffer AE directly onto the membrane.

#### Digestion of gDNA with restriction enzymes

A quantity of 500 ng gDNA, 1 µl of FastDigest restriction enzyme (BamHI, EcoRI, or HindIII; Life Technologies), and 1 µl of the FastDigest Green Buffer supplied with the enzyme in a total volume of 10 µl were incubated at 37°C for 30 min. Digested DNA was loaded onto a 1% w/v agarose gel containing TBE buffer and DNA electrophoresis was performed for 1 h at 200 V. Bands in gels were stained with Ethidium Bromide.

#### Polymerase chain reaction

Polymerase chain reaction (PCR) was carried out using 100 ng of gDNA, 0.5 μM of the primers I+T+S+1 (ITS1) and ITS4 as described by White et al. (1990), and 1× Phusion® High-Fidelity PCR Master Mix with HF Buffer (M0531; NEB) in a final volume of 20 μl. Amplification was performed in a T100 Thermal Cycler (Bio-Rad), under the following conditions: 98°C for 2 min, 25 cycles of (98°C for 10 s, 47, 55, or 60°C for 30 s, and 72°C for 25 s), and a final extension at 72°C for 10 min.

#### PCR amplification of ITS region for sequencing

PCR was carried out using 250 ng of gDNA, 0.5 μM of ITS1 and 0.5 μM of ITS2 primers, and 1× Phusion® High-Fidelity PCR Master Mix with HF Buffer in a final volume of 50 μl, under the following conditions: 98°C for 2 min, 35 cycles of (98°C for 10 s, 47, 55, or 60°C for 30 s, and 72°C for 25 s), and a final extension at 72°C for 10 min. The 648 bp product was analysed on a 2% agarose gel and then extracted using QIAquick gel extraction kit (28704; Qiagen). Purified DNA was sequenced using ITS1, ITS2, ITS3, and ITS4 primers (White et al. 1990) at the Sanger Sequencing Facility, The Centre for Applied Genomics (TCAG), Toronto.

#### Determination of the species of the Aspergillus sample

PCR was carried out using 100 ng of gDNA, 0.5 μM of each primer (see Table 1), and 1× Phusion® High-Fidelity PCR Master Mix with HF Buffer (M0531; NEB) in a final volume of 20 μl. Amplification was performed in a T100 Thermal Cycler (Bio-Rad), under the following conditions: 98°C for 2 min, 25 cycles of (98°C for 10 s, 55°C for 30 s, and 72°C for 25 s), and a final extension at 72°C for 10 min.

**Table 1.** Species-specific PCR primers for amplification of partial sequence of the calmodulin gene in *A. niger* or *A. tubingensis.*

Region	Name	Sequence (5′–>3′)	Reference
Calmodulin	NIG1 (Forward)	TCCGTAGGTGAACCTGCGG	Susca et al. (2007)
Calmodulin	NIG2 (Reverse)	GCTGCGTTCTTCATCGATGC	
Calmodulin	TUB1 (Forward)	GCATCGATGAAGAACGCAGC	
Calmodulin	TUB2 (Reverse)	TCCTCCGCTTATTGATATGC	

### Spores preparation

The fungus was cultivated in a 3.9% (w/v) PDA plate and incubated at 30°C for 7 days to produce an adequate number of spores (Bosshard et al. 1996). Spores from 1-week-old cultures were washed with 10 ml of sterile distilled water. The suspensions were then ﬁltered with sterile glass wool and stored at 6°C. The number of spores was determined with a haemocytometer (Mulligan and Kamali 2003).

### Solubilisation experiments

The tests were conducted in triplicates in sterilised 250 ml Erlenmeyer ﬂasks containing 100 ml of NBRIP medium supplemented with different weight/volume percentages (1%, 2%, 4%, or 8%) of pre-sterilised RP. Flasks were inoculated with 1 ml of spore suspension and were then incubated on an orbital shaker for 18 days at 28°C, 150 rpm. In parallel and under identical growth conditions and RP concentrations, the control experiments were conducted with fresh medium without fungi. In all the tests, a 5–8-ml sample was taken aseptically from the flasks at predetermined intervals and analysed for pH, organic acids concentration, P concentration, and acid phosphatase and then the volume in each flask was topped up to 100 ml with sterile NBRIP medium. At the end of incubation period, the RP from 1% treatment was chosen for XRD and XRF analysis.

### Analytical techniques

Immediately following each sampling, samples were filtered through 0.22 μm nylon membrane filters (Millipore) to ensure that the suspensions were particle-free. The pH of each sample was measured using a pH meter (Jenway, 3520), which was calibrated periodically using buffer solutions of pH 4.01 and pH 7.00 (Merck). Solubilised P concentration was determined by the phosphomolybdate method (Murphy and Riley 1962). The acid phosphatase activity in the culture medium used for P solubilisation experiment was determined by the hydrolysis of *p*-nitrophenyl phosphate method described by Tabatabai and Bremner (1969). Culture ﬁltrate was incubated with *p*-nitrophenyl phosphate and modiﬁed universal buffer. Hydrolysis reaction of *p*-nitrophenyl phosphate by phosphatase was terminated an hour later by adding a solution of 0.5 M CaCl_2_ and 0.5 M NaOH. The mixture was centrifuged and the yellow colour of supernatant was measured at 410 nm. Organic acids were determined using a high performance liquid chromatography (HP 1100 series) device equipped with UV detector on Aminex HPX-87H, 300 mm × 7.8 mm (Bio-Rad) column with microguard cation H (Bio-Rad), pre-column attached, 5 mM H_2_SO_4_ as the mobile phase, flow rate of 0.5 ml/min at ambient temperature, and the detection was carried out at 210 nm. The acid concentrations of samples were calculated from the integrated areas of the peaks using external standards. Prior to analysis, the samples were filtered with 0.2 µm nylon membrane syringe filter (Titan) to protect the column from being clogged by fine particles in the samples. This method was adapted from Bosshard et al. (1996).

### Statistical analyses

All experimental data were subjected to ANOVA procedure and differences between means were determined using Tukey’s test at 5% confidence level. The statistical analysis of the data was performed with the Statistix statistical software package (Analytical Software 2007).

## Results

### Isolation and identification of fungal isolate

**Figure 1.**
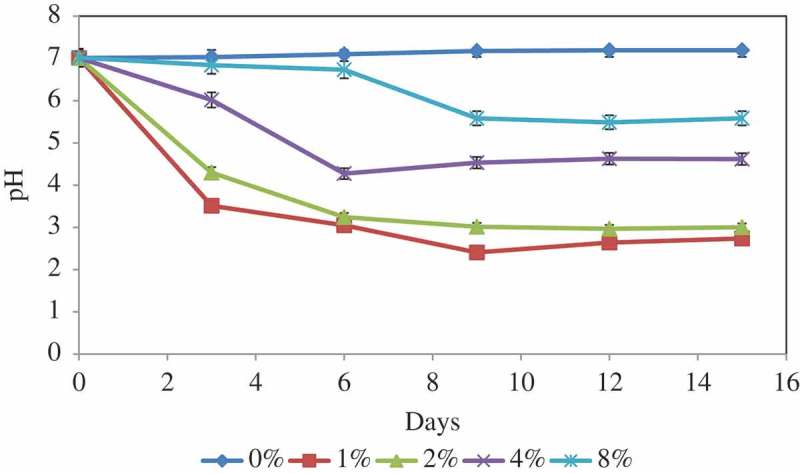
Variations of culture pH at various RP concentrations during incubation period. Vertical bars denote standard deviation, *n* = 3.

Mine soil samples were collected from Dalir phosphate mine at 36°19′33″N 51°05′35″E in Chalous, Mazandaran, Iran. Over 25 random points were chosen and soil samples were collected using sterile techniques. Following inoculation, growth, and selection of fungal isolates, the fungal strain SANRU, which showed the biggest clear zone around colony, was selected for further studies. In order to genetically identify the species of the selected SANRU strain, the mycelium was cultured, and harvested, before extraction of gDNA. To assure the successful extraction of gDNA and its suitability to serve as template in PCR reactions, gDNA was digested by different restriction endonucleases. Using primers sets (ITS1 and ITS4) that amplify the ribosomal RNA gene sequences of broad fungal taxa (Toju et al. 2012), the PCR was amplified ITS1 (282 nt), ITS2 (385 nt), and the entire ITS (648 nt) region of the SANRU strain. The PCR product of the entire ITS region was subjected to gDNA sequencing using the ITS primers; sequencing results were assembled and then BLAST searches were conducted to the Nucleotide database of the National Center for Biotechnology Information (NCBI; http://blast.ncbi.nlm.nih.gov/). The nucleotide blasted ITS region of SANRU strain showed 100% similarity to both *A. niger* and *A. tubingensis*; the two species are morphologically highly similar. The DNA sequence was then deposited in the GenBank nucleotide sequence data library under the accession number KT222864. To overcome the uncertainty, we performed a specific PCR assay identification targeting DNA sequence of the calmodulin gene which was previously proven to efficiently differentiate between the two species since it contains some species-specific diagnostic traits (Susca et al. 2007). Table 1 shows the species-specific PCR primers for amplification of a partial sequence of the calmodulin gene in *A. niger* or *A. tubingensis*. Primers N1G1/N1G2 amplify only DNA belonging to *A. niger* strains and the primer pair TUB1/TUB2 amplify only DNA belonging to *A. tubingensis*, confirming their specificity (Susca et al. 2007). When subjected to PCR amplification of a partial sequence of the calmodulin gene using the above primer pairs, the specific primer pair TUB1/TUB2 amplified a 505-bp fragment, whereas the NIG1/NIG2 set failed to amplify any region, strongly suggesting that the SANRU strain belongs to the species *tubingensis* (Figure 1).

### pH changes in RP solubilisation

**Figure 2.**
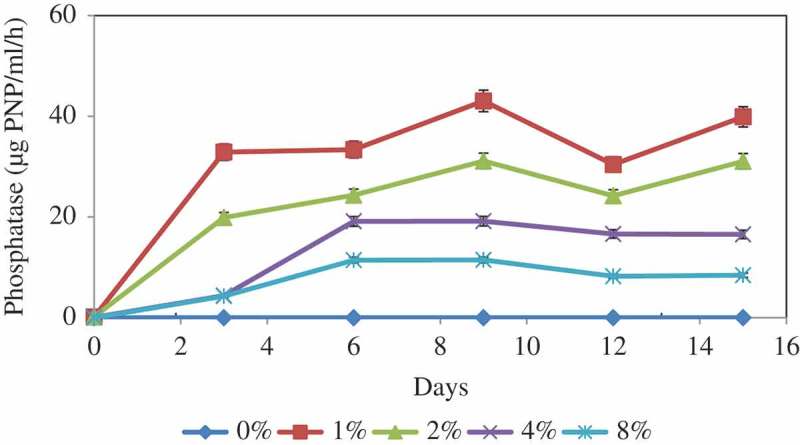
Phosphatase production at various RP concentrations during incubation period. Vertical bars denote standard deviation, *n* = 3.

The pH changes in medium culture at different concentrations of RP over time during the solubilisation processes were as shown in Figure 2. In general, pH changes were less drastic with increasing RP concentrations. Maximum pH decrease occurred on day 9 of incubation for all RP concentrations. Therefore, the initial pH which was around 7 for all the treatments dropped to 2.4, 3.01, 4.53, and 5.58 for 1%, 2%, 4%, and 8% RP concentrations, respectively. After this incubation period, the pH for different RP concentrations remained nearly constant. The higher concentration of RP in the culture medium induced more buffering capacity; therefore, change was less drastic in medium with higher concentration of RP. The pH level in the control samples using fresh medium was almost constant during the leaching process due to lack of fungal activity (data not shown).

### Organic acid production

Since one of the major mechanisms for the solubilisation of RP by microorganisms is acidolysis, the type and concentration of organic acids produced in the RP solubilisation was determined on day 9. Table 2 shows the types and concentrations of organic acids produced in the RP solubilisation. In this study, the fungus produced only citric and oxalic acids in the presence of RP. The quantification studies determined an inverse relationship between the concentrations of citric and oxalic acids and the RP concentration and maximum organic acid concentration was achieved at 1% RP concentration. As Table 2 shows, the fungus produced more citric acid (19.4 mM) than oxalic acid (10.27 mM).

**Table 2.** Organic acid production by *A. tubingensis* SANRU at various RP concentration on day 9 of incubation time.

RP concentration	Citric acid (mM)	Oxalic acid (mM)
1	*19.4 A	10.27 A
2	12.36 B	4.1 B
3	7.5 C	1.26 C
4	3.4 D	0 C

RP: Rock phosphate. *Values are means of three replicates. Means values within a column followed by the same letter are not significantly different (*p* < 0.05) based on Tukey’s test.

## Phosphatase activity

**Figure 3.**
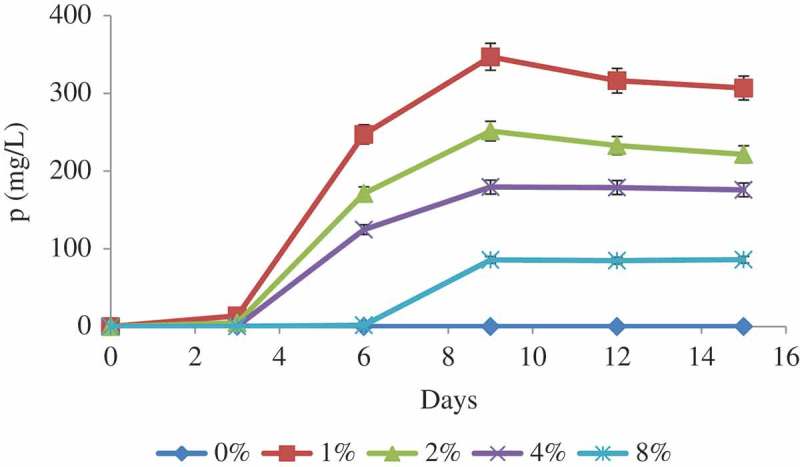
Solubilisation of P by *A. tubingensis* SANRU at various RP concentrations during incubation period. Vertical bars denote standard deviation, *n* = 3. Means with different letters are signiﬁcantly different from each other (*p* < 0.05) according to the Tukey’s test.

Acid phosphatase production by *A. tubingensis* SANRU at various RP concentrations is shown in Figure 3. Like pH and organic acid production, the phosphatase secretion follows clear trend with the variation of RP concentration. While the maximum phosphatase activity was recorded for 1% RP concentration, this was followed by 4% at the end of 9 days of incubation. Phosphatase does not act directly on inorganic P solubilisation, but phosphatase activity may participate in lowering the pH of the culture medium by dephosphorylation and the production of acids.

### Fungal solubilisation of RP

**Figure 4.**
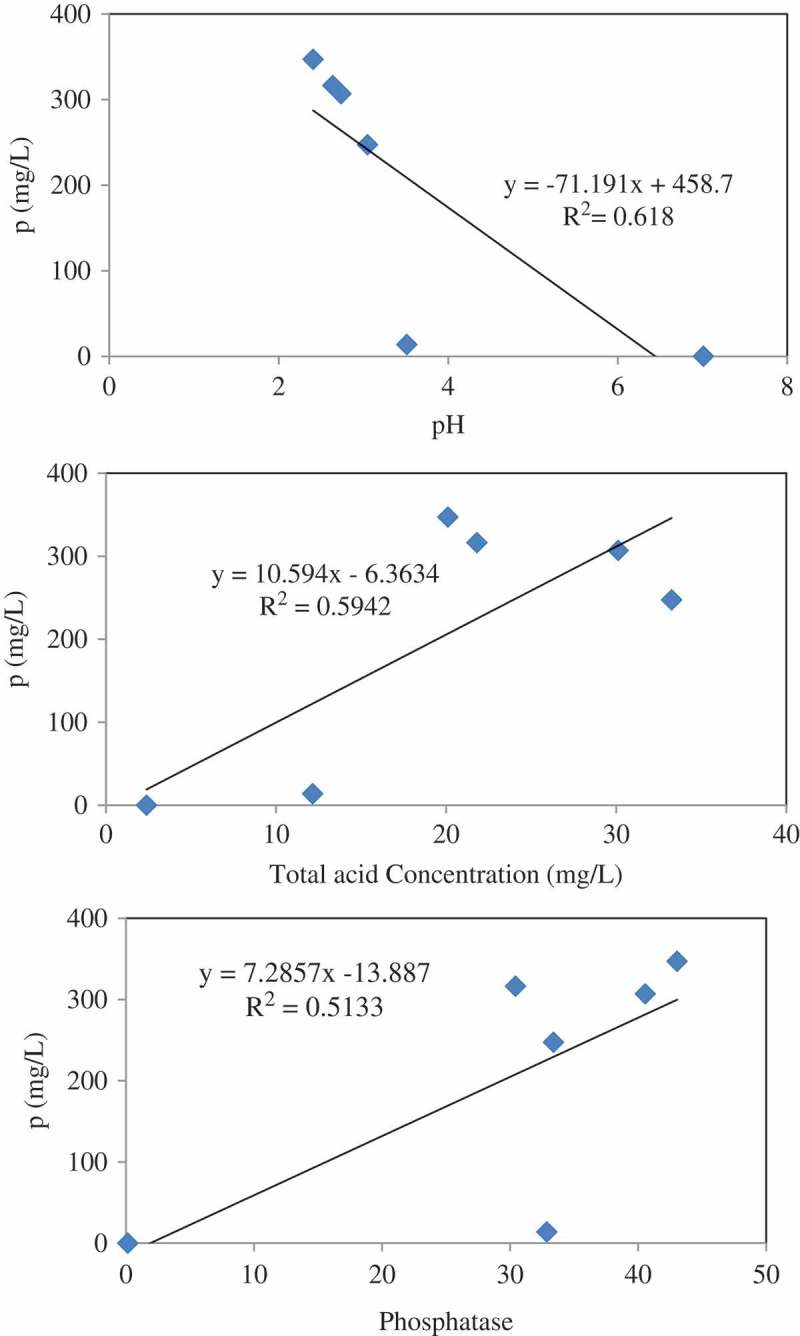
Relationship between pH (a), organic acid (b) and acid phosphatase (c) of bacterial culture and solubilised P. Each value represents the mean of three replicates with standard deviation shown by error bars.

**Figure 5.**
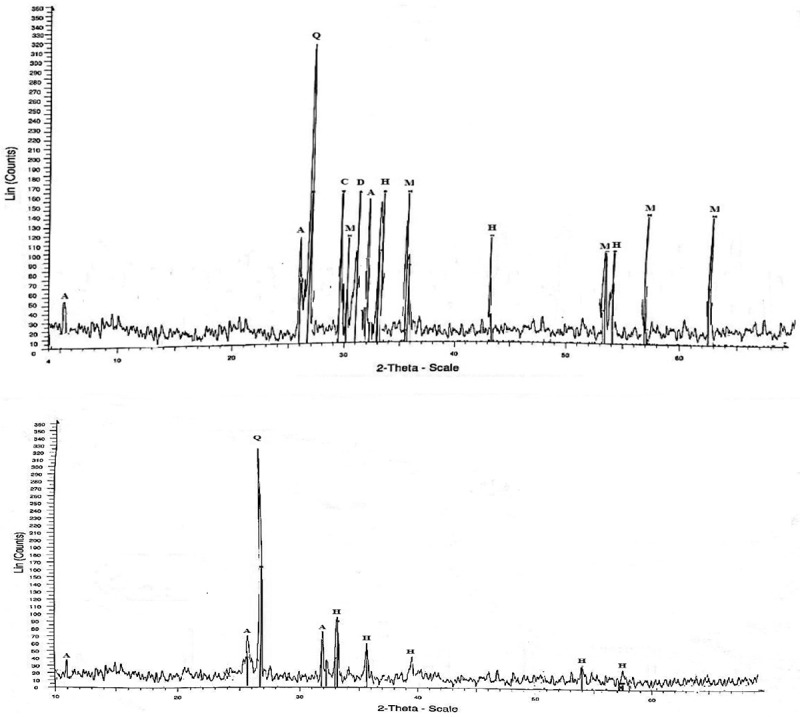
XRD diffractogram of RP of control (a) and solubilised by *A. tubingensis* SANRU (b), respectively, at the incubation period. Letter designations: A, apatite; C, calcite; H, haematite; D, dolomite; M, magnetite; Q, quartz.

Phosphorus solubilisation by *A. tubingensis* SANRU at various RP concentrations is shown in Figure 4. The P concentration in all treatments significantly increased at day 9, and then remained constant. The highest P solubilisation averaged 347 mg/l in the presence of 1% RP concentration. Generally, solubilisation of P decreased when the concentration of RP increased. The P solubilisation from RP by microorganisms is apparently associated with acidification and enzymatic reactions (Park et al. 2011), so regression analysis was carried out to determine the significance of the relationship between pH, organic acid concentration, and phosphatase activity with P solubilisation at 1% RP concentration on day 9 of incubation time. The regression analysis between solubilised P and pH (*R*^2^ = 0.61, Figure 5(a)), organic acid (*R*^2^ = 0.59, Figure 5(b)) and acid phosphatase activity (*R*^2^ = 0.51, Figure 5(c)) showed a signiﬁcant linear relationship (*p* < 0.001). These data show that pH reduction is most effective mechanisms in P solubilisation through fungal action. Compared with other reported literatures, the values obtained from the present study are promising (Table 3).

**Table 3.** Comparison of P solubilisation from rock phosphate in the present study and with those obtained in the literatures.

Fungal strain	Solubilised P (mg/l)	References
*Aspergillus tubingensis* SANRU	347.07	Present study
*Aspergillus niger*	382	Schneider et al. (2010)
*Aspergillus niger*	306	Schneider et al. (2010)
*Aspergillus niger*	281	Schneider et al. (2010)
*Aspergillus tubingensis*	277	Achal et al. (2007)
*Aspergillus niger*	147	Mendes et al. (2014)
*Aspergillus niger*	127	Mendes et al. (2014)
*Aspergillusawamori* S19	81	Jain et al. (2012)

### XRD and XRF analysis

The XRD pattern of 1% RP before and after solubilisation by *A. tubingensis* SANRU is presented in Figure 5. Based on the X-ray diffractogram (XRD), the mineralogical constituents before solubilisation were quartz, hydroxylapatite, magnetite, hematite, calcite, and dolomite. Interestingly, the RP residue after 18 days solubilisation only contains quartz, hematite, and hydroxylapatite. The XRD pattern also showed a significant reduction in the amount of P in the RP residues compared to the RP before solubilisation. The XRF chemical analysis (Table 4) indicated that RP contained 10.3% P_2_O which dropped to 7.3% after solubilisation. This sudden change indicated that solubilisation by the fungus had a significant impact in changing RP composition.

**Table 4.** XRF elemental analysis of RP before and after bio-solubilisation.

Composition	wt%		Composition	wt%	
	Before	After		Before	After
P_2_O_5_	10.7	7.1	Al_2_O_3_	1.3	2.7
Na_2_O	0.5	0.06	K_2_O	0.28	1.6
TiO_2_	0.73	0.72	SiO_2_	24.4	24.3
MgO	1.4	1.2	CaO	21.5	13.0
Cl	0.25	0.21	LOI^§^	7.34	21.0
Fe_2_O_3_ + FeO	30.7	26.8			

RP: Rock phosphate; XRF: X-ray ﬂuorescence. ^§^Loss on ignition.

## Discussion

RPs are considered as valuable alternatives to phosphorus fertilisers. However, most of the RPs are low grade and insoluble (Xiao et al. 2008). Currently, due to the shortage of natural ores as well as environmental considerations, solubilisation of RPs using microorganisms has gained increasing attention. In the present study, a fungal strain, SANRU, which has shown remarkable P-solubilising activity on NBRIP agar, was obtained from a phosphate mine in Iran. To identify strain SANRU, we used ITS primers first, but due to the lack of differentiation between the two types of *A. niger* and *A. tubingensis* by the primer, the calmodulin-based PCR assay was used for the identification of this strain (Susca et al. 2007). Phosphorus biosolubilisation efficiency of this strain was tested in culture medium. The ability of microorganisms (bacteria and fungi) to solubilise P from solid materials occurs via acidification, complexation, and enzymatic reactions (Park et al. 2011). One of the potential mechanisms for phosphate solubilisation might be acidification of the medium during the growth of fungi due to the following four processes (Burgstaller and Schinner 1993): (1) the excretion of protons via the proton translocating plasma membrane ATPase; (2) the absorption of nutrients in exchange for protons; (3) the excretion of organic acids; and (4) the production of carbon dioxide by the respiratory activity of the fungus. The acidification of hydroxylapatite occurs by the following reaction which results in the formation of more soluble phosphates:

$$Ca_{5}{\left(PO_{4}\right)}_{3}\left(OH\right)+4H^{+}=5Ca^{2+}+3HP{O_{4}}^{2-}+H_{2}O$$

By decreasing pH, the solubility of calcium phosphate increases (Bashan et al. 2013).

Organic acid production plays an important role in the acidification of the culture medium and subsequently in P solubilisation. Organic acids, by acidolysis of their negative charge or through their metal complexation properties, affect P solubilisation. There is no information on organic acid production by *A. tubingensis* in the presence of RP. In the culture medium, only citric and oxalic acids were detectable. This was in agreement with Magnuson and Lasure (2004) who mentioned that during the metabolism of sucrose, noticeable amounts of pyruvic acid could enter in tricarboxylic acid cycle. As mentioned earlier, the organic acid concentration decreased by increasing RP concentration. This may suggest that the fungus produced constant organic acid in the presence of different concentration of RP; hence, at higher RP concentrations, the consumption of organic acids increased and leads to less RP solubilisation than lower RP concentrations. Our results also showed that citric acid was produced in higher amounts than oxalic acid. This finding is in agreement with some research, which found low pH (˂3) favour for citric acid production by fungi but not oxalic acids (Burgstaller and Schinner 1993; Bosshard et al. 1996). On the other hand, according to the reaction below oxalic acid precipitates Ca as calcium oxalate, which has a low solubility (*K*_sp_ = 2:74 × 10^−^^11^).

$$Ca_{5}{\left(PO_{4}\right)}_{3}OH+5H_{2}C_{2}O_{4}=5CaC_{2}O_{4}+3H_{2}PO_{4}+H_{2}O$$

Maximum P was removed from RP at 1% concentration, which it may be due to reabsorption of a part of the solubilised P since the fungus has the capacity to solubilise a specific quantity of P which is in equilibrium with fixed P (Duponnois et al. 2006). Results also showed that the P solubilisation in all treatments increased significantly on day 9, thereafter decreased slightly and then remained constant. This fact may be attributed to the availability of soluble phosphorus, which had an inhibitory effect on further RP solubilisation, or the depletion of carbon source might have limited both the production of organic acids and microbial activity. The effect of RP concentration on the P solubilisation can be due to the different amounts of organic acids available in the medium and the difference in pH (Table 2; Figure 2). Although there was only a slight increase (5.32 mg/l after 14 days) in the content of soluble P under the control conditions, no significant change was observed.

## Conclusions

The results of the present investigation indicate that *A. tubingensis* SANRU is effective in phosphate solubilisation. However, further trials are needed to determine the capability of the organisms for P solubilisation in pot experiments or under filed condition.
